# Primary vaginal signet ring cell carcinoma

**DOI:** 10.1259/bjrcr.20220050

**Published:** 2022-11-22

**Authors:** Xiao-Shan Huang, Shu-Feng Fan, Jian-Xia Xu, Yong-Jie Xu, Xia Song, Jun-Yi Xiang, Li-Ming Xue

**Affiliations:** 1 Department of Radiology, The Second Affiliated Hospital of Zhejiang Chinese Medical University, Hangzhou, China; 2 Department of Computed Tomography and Magnetic Resonance Imaging, Handan First Hospital, Handan, China

## Abstract

Primary vaginal cancer is rare, accounting for only 2% of all gynecological malignant tumors. Primary vaginal cell carcinoma is mainly squamous cell carcinoma, accounting for about 90%, and adenocarcinoma only accounts for 8–10%. Primary signet ring cell carcinoma of vagina is rare and has not been reported in the literature. This paper reports a case of signet ring cell carcinoma in vagina.

## Case presentation

**Figure 1.**
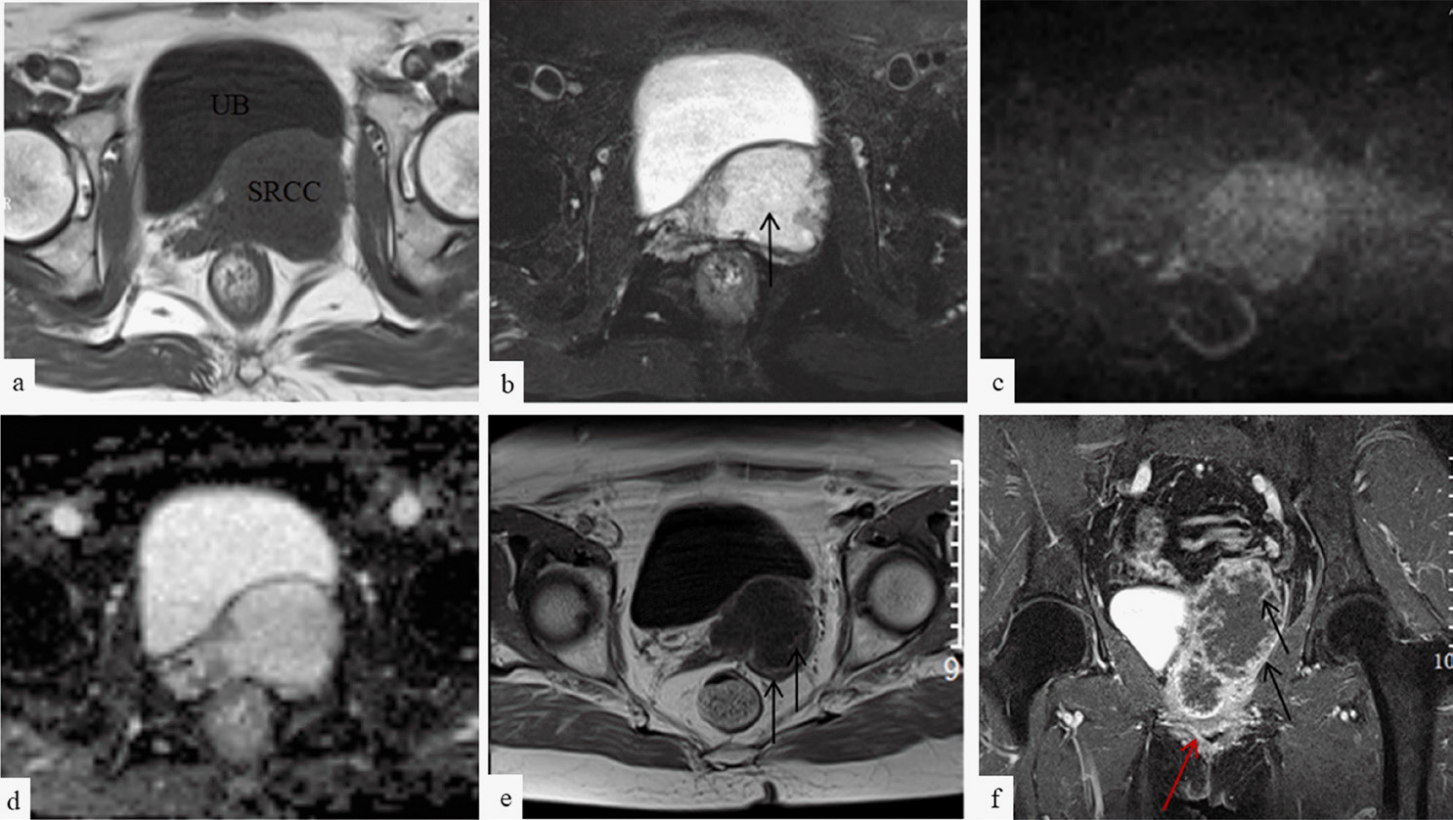
MRI image shows vaginal signet ring cell carcinoma. Axial *T*_1_ weighted MR image (**a**) shows a lesion in the vagina. Axial T2 fat suppression image (**b**) shows heterogeneous high signal intensity and cystic change of tumor (black arrow). Diffusion-weighted MR image (**c**) and ADC map (**d**) show unrestricted diffusion in the tumor. Arterial phase image (**e**) shows mild enhancement of the margin and grid-like enhancement in the interior of the tumor (black arrows). Delay phase image (**f**) shows further enhancement of the margin and the interior of the tumor (black arrows). Vagina (red arrow). ADC, apparent diffusion coefficiemnt; SRCC, Signet ring cell carcinoma; UR, Urinary bladder.

**Figure 2.**
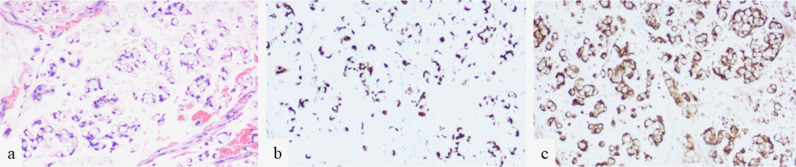
Microscopic examination with high power (×400) (**a**) reveals signet ring cells. Immunohistochemistry showed positivity for CDX-2 (**b**), and villin (**c**).

**Figure 3.**
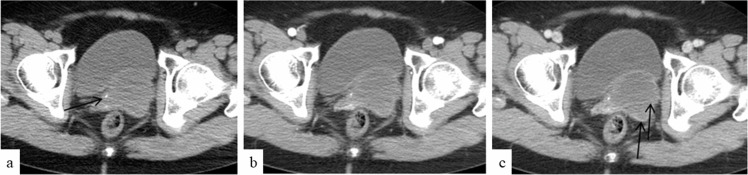
CT image shows vaginal signet ring cell carcinoma. CT image (**a**) shows the tumor with heterogeneously slightly low density and spotty calcification of the margin (black arrow). Arterial phase image (**b**) shows mild enhancement of the margin in the tumor. Venous phase image (**c**) shows further enhancement of the margin and mild grid-like enhancement in the interior of the tumor (black arrows).

**Figure 4.**
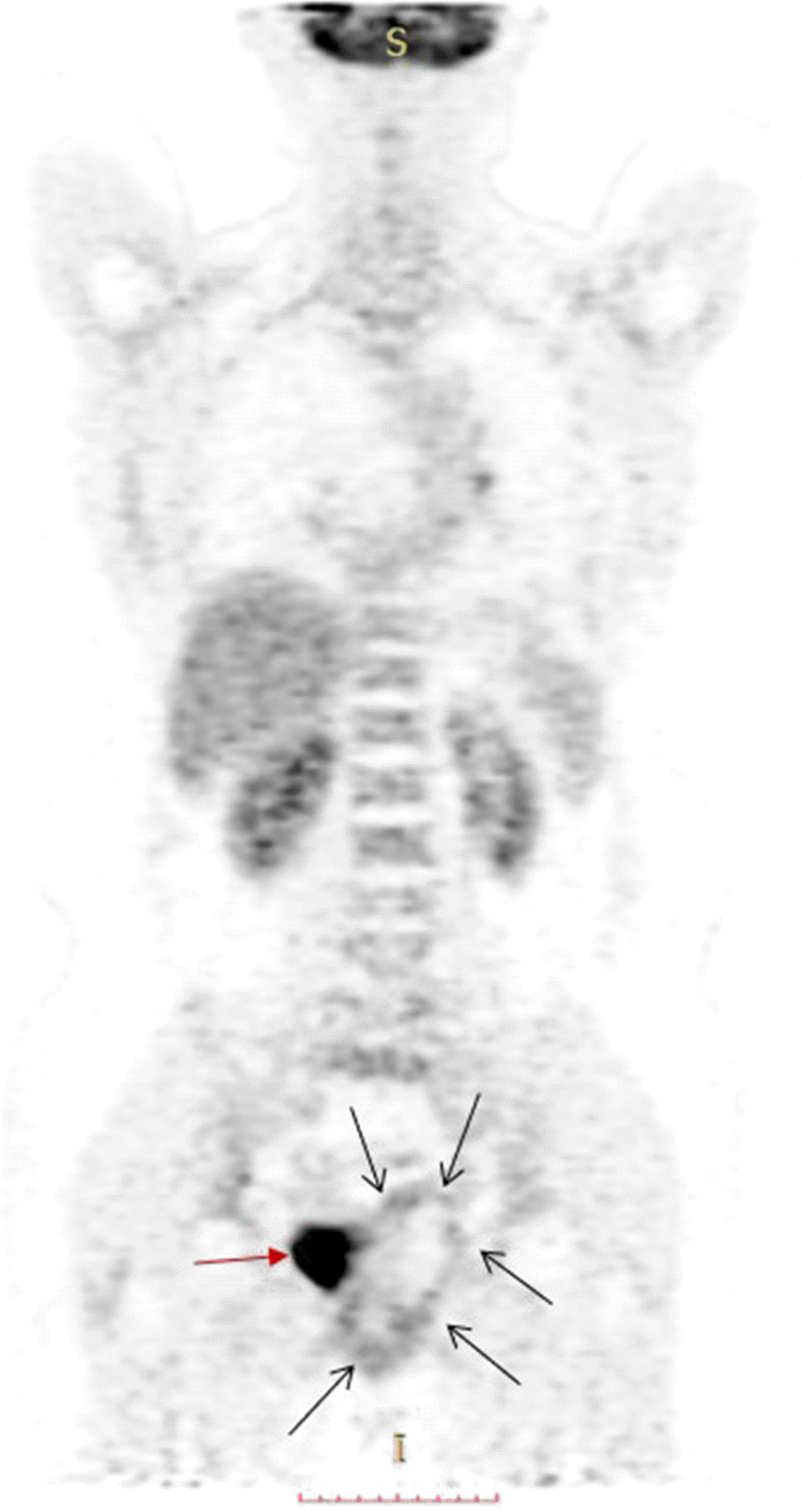
PET-CT image shows a lesion in the vagina (black arrows). Urinary bladder (red arrow）. PET-CT, positron emission tomography-computed tomography.

A 65-year-old female was admitted to the Second Affiliated Hospital of Zhejiang Chinese Medical University in April 2019, and she reported a vaginal bleeding after sexual intercourse in December 2018. Pelvic ultrasound demonstrated a pelvic hematoma, and pharmaceutical conservative treatment was administrated. However, she had intermittent vaginal bleeding in the following three months.^1^ Considering that the patient previously underwent total hysterectomy for uterine prolapse, the uterine pathologic specimen was reviewed. The patient had neither cervical and endometrial dysplasia or carcinoma *in situ* (CIS) nor a history of exposure to diethylstilbestrol (DES), and she was negative for human papilloma virus (HPV). Physical examination revealed a palpable mass (6.0 × 6.0 cm) in the pelvic cavity, which was hard in texture and poor in mobility, without significant tenderness. A small amount of dark-red secretions were visible within the vagina, and the anterior vaginal wall was hard and presented with irregular nodules. Vaginal neoplastic lesion was considered, and tumor index test and pelvic enhanced MRI were performed. Laboratory examination revealed elevation of CA125 (63.4 U ml^−1^) and cytokeratin 19 fragment (3.17 U ml^−1^), and other normal tumor indices. On pelvic MRI, a mass with a predominant cyst component (around 5.9 × 4.5 × 8.9 cm in size), which enveloped the left lower ureter and closely adhered to the posterior bladder wall and the rectum, was found within the vagina. The mass was hypointense signal on *T*_1_ weighted imaging (*T*_1_WI) (Figure 1a), heterogeneously hyperintense signal on *T*_2_ weighted imaging fat suppression (*T*_2_WI-FS) (Figure 1b), and slightly hyperintense signal on diffusion-weighted imaging (DWI) (Figure 1c) with increased apparent diffusion coefficient (ADC) (Figure 1d). On enhanced MRI, the arterial phase showed mild enhancement of the margin and the mild grid-like enhancement of the interior of the mass with no enhancement in the cystic cavity (Figure 1e); while the delayed phase showed further enhancement of the margin and the interior of the mass (Figure 1f). A vaginal malignancy was suggested, and whether it was a metastasis needed to be validated. To confirm the pathology of the vaginal tumor, biopsy of the vaginal mass was suggested and approved by the patient. Pathologic findings showed that the mass was nodular segmented by fibrous tissues and rich in mucin within the stroma. The cells were in medium-size, and the majority of them showed clear cytoplasm rich in mucin and partial acidophilic cytoplasm. In addition, the nuclei were hyperchromatic and had a signet ring appearance after squeezing to one side with visible mitosis (Figure 2a). Immunohistochemistry showed CDX-2 (+) (Figure 2b), Villin (+) (Figure 2c), CK7 (+), GATA-3 (+), MUC-1 (weakly +), P53 (+), KI67 10%+ (CA125-, ER-, PR-). Combining the pathology and immunohistochemistry, vaginal signet ring cell carcinoma was diagnosed, while gastrointestinal tumor metastasis could not be excluded. Since primary vaginal tumor is rare, systemic examination is required to exclude metastasis. Vaginal signet ring cell carcinoma usually originates from gastrointestinal signet ring cell carcinoma and sometimes from breast and bladder signet ring cell carcinomas. Therefore, the patient was scheduled for gastroscopy, breast ultrasound, and abdominal-enhanced CT. No significant abnormalities were found on both the gastroscopy and breast ultrasound. The uterus and bilateral adnexa were absent on abdominal CT. Besides, an irregular mass skewed to the left and sized around 5.9 × 4.5 × 8.9 cm was found within the vagina, presenting with heterogeneously slightly low density and spotty calcification of the margin. The left lower ureter was enveloped by the mass, and part of the mass was closely adhered to the posterior bladder wall and the rectum (Figure 3a). On enhanced CT scan, mild-to-moderate enhancement of the margin and mild grid-like enhancement in the interior of the mass were demonstrated (Figure 3b–c). There was no significant abnormality in the gastrointestinal tract or other abdominal organs. Vaginal malignancy was reported, and metastasis could not be excluded. Considering all the aforementioned evidence collectively, primary signet ring cell carcinoma was highly suspected. Positron emission tomography-computed tomography (PET-CT) was given to confirm whether the tumor was primary or metastatic, and a local malignant tumor within the vagina with no any other neoplastic lesion in other body sites was reported (Figure 4). Combining the pathology, immunohistochemistry, and imaging results, primary vaginal signet ring cell carcinoma was diagnosed. The patient then underwent laparoscopic exploration under general anesthesia, showing absence of tumor in the abdominal organs (*e.g.* liver, gallbladder, pancreas, diaphragm, stomach, and intestine) or the upper abdominal peritoneum. Besides, adhesions were found between intestinal wall/peritoneum and vaginal stump/bladder wall. After the adhesions were separated, an extraperitoneal mass, which was approximately 9.0 × 5.0 × 5.5 cm in size with a hard texture, was found as protruding from the vaginal stump to the left posterior bladder. The left ureter was enveloped by the mass, and the posterior margin of the mass was tightly adhered to some rectum. Neither the uterus nor bilateral adnexa were observed. The surgery scope required to be expanded, but the patient family refused.

## Discussion

Primary vaginal cancer is rare. Most of these tumors are squamous cell carcinomas, while others are adenocarcinoma, sarcoma and melanoma.^2^ About 5% of primary malignant vaginal tumors are adenocarcinoma. Several subtypes of vaginal adenocarcinoma have been reported in the literature, including clear cell carcinoma, endometrioid carcinoma, serous carcinoma, and mucinous carcinoma.^3^ Signet ring cell carcinoma of vagina has not been reported in the literature. Clement and Dhorepatil suggest that focal signet ring cells can be seen in vagina, but neither author has reported a definite case of signet ring cell carcinoma of vagina.^4,5^ According to the international classification standard of cervical adenocarcinoma,^6^ under the background of common adenocarcinoma, signet ring cell carcinoma is diagnosed when the number of signet ring cell is ≥50%.

Primary vaginal tumors are rare, so extensive systemic examination is needed to rule out metastasis before diagnosis of primary signet ring cell carcinoma of vagina. Vaginal signet ring cell carcinoma usually originates from gastrointestinal signet ring cell carcinoma and sometimes from breast and bladder signet ring cell carcinomas. Immunohistochemical staining showed that CDX-2, Villin and MUC-1 were positive, suggesting a high possibility of gastrointestinal metastasis. Subsequently, the patient underwent gastroscopy, breast ultrasound, abdominal-enhanced CT and PET-CT, and no tumors were found in gastrointestinal tract and other parts of the body. The final diagnosis was primary signet ring cell carcinoma of vagina.

The pathogenesis of signet ring cell carcinoma of vagina is unclear. In 89% of cases, vaginal cancer may be a high-risk HPV-related disease.^1^ There are also some literatures that hysterectomy is related to the occurrence of vaginal cancer. In the study of 91 patients with vaginal cancer conducted by Hiniker et al., 46% of patients have a history of hysterectomy,^7^ and in the study of Mariana Lima et al., 80% of patients with vaginal adenocarcinoma have a history of hysterectomy.^8^ At the same time, Clement’s case report shows that adenocarcinoma and focal signet ring cells were found in vagina 15 months after hysterectomy.^4^ The patient in our article underwent hysterectomy for uterine prolapse 3 years ago. At that time, the pathological result was benign, the patient had no history of exposure to DES and HPV was negative. Therefore, it is speculated that the occurrence of signet ring cell carcinoma of vagina in this case may be related to hysterectomy.

The overall vaginal tumor reported here was slightly hypointense signal on *T*_1_WI, heterogeneously hyperintense signal on *T*_2_WI, and slightly hypointense signal on DWI with significant ADC value elevation. Additionally, the tumor presented with enhancement of the margin and grid-like enhancement in the interior of the mass. A previous study demonstrated that the hyperintense signal area on *T*_2_WI might indicate a mucin-producing tumor.^9^ Consistently, the MRI findings suggested that the tumor was rich in mucin. Signet ring cell carcinoma is a type of mucinous adenocarcinoma rich in mucin. Tumor imaging features, such as MRI manifestations, fail to make a direct diagnosis of signet ring cell carcinoma but provide some indications. Moreover, CT images are capable of showing spotty calcification within a lesion, which is also a manifestation of signet ring cell carcinoma. In the study of Yang et al,^10^ the calcification in signet ring cell carcinoma was considered as the deposition of calcium salts in the intra- or extracellular mucus lakes, which presented as punctate, miliary, or nodular calcific foci on CT images. Presently, the cause of calcification remains elusive, and it is speculated as correlated to the mucoricin within and alkaline environment of the mucus lakes. In terms of the MRI manifestations, signet ring cell carcinoma usually is slightly hypointense signal on *T*_1_WI and heterogeneously hyperintense signal on *T*_2_WI due to the large amount of mucous component. In addition, it is iso-to-hyperintense signal on DWI given the increase in the interstitial space of the tissue. Both enhanced CT and MRI images showed enhancement of the margin and grid-like enhancement in the interior of the tumor, which is also reflective of the mucous component. The grid-like enhancement in the interior of the tumor is considered a result of the presence of some interstitial and vascular components.^11^

The differential diagnosis of signet ring cell carcinoma of vagina includes metastatic carcinoma, squamous cell carcinoma, clear cell carcinoma, melanoma, and sarcoma, among which metastatic carcinoma needs to be differentiated first. Signet ring cell carcinoma usually originates from gastrointestinal, manifesting diffuse thickening of the gastrointestinal wall with visible spotty or nodular calcification. Additionally, it presents with layered delayed enhancement after enhanced scan. In the case reported here, spotty calcification was found within the lesion on CT scan and delayed enhancement was indicated after enhanced scan, similar to gastrointestinal signet ring cell carcinoma.^10–12^ However, the present case showed a mass with a predominant cystic component due to the copious mucin, which is different with the gastrointestinal signet ring cell carcinoma. In case of suspicion for vaginal signet ring cell carcinoma, it is important to first identify whether there is a primary tumor. If no primary tumor is found, primary vaginal signet ring cell carcinoma will be considered; otherwise, imaging and pathology are further required to confirm the diagnosis. Because primary vaginal cancer is rare and complicated to treat, it should follow the principle of individualization, and the treatment plan should learn from the treatment methods of cervical cancer.^1^ It is reported^13^ that chemotherapy combined with radiotherapy and surgery can prolong the survival time of cervical signet ring cell carcinoma, especially systemic chemotherapy can control distant metastasis and improve the curative effect.

## Conclusion

The morphology and immunohistochemistry of primary signet ring cell carcinoma of vagina are similar to those of gastrointestinal signet ring cell carcinoma, and it is difficult to diagnose it only by pathology and immunohistochemistry. The final diagnosis must be combined with imaging, pathology, and immunohistochemistry.

## Learning points

- Signet ring cell carcinoma is highly malignant, mainly occurring in gastrointestinal tract and rarely in vagina.
- MRI can well reflect the mucous components of tumors and has certain diagnostic value for mucinous tumors.
- The diagnosis of primary signet ring cell carcinoma of vagina remains a challenge. The final diagnosis must be combined with imaging, pathology, and immunohistochemistry.

## References

[1] Rajaram S, Maheshwari A, Srivastava A . Staging for vaginal cancer. Best Pract Res Clin Obstet Gynaecol 2015; 29: 822–32. doi: 10.1016/j.bpobgyn.2015.01.006 25847318

[2] Gadducci A, Fabrini MG, Lanfredini N, Sergiampietri C . Squamous cell carcinoma of the vagina: natural history, treatment modalities and prognostic factors. Crit Rev Oncol Hematol 2015; 93: 211–24. doi: 10.1016/j.critrevonc.2014.09.002 25476235

[3] Tatsumi K, Schlappe B, Everett EN, Gibson PC, Mount SL . Primary vaginal mucinous adenocarcinoma of intestinal type, associated with intestinal metaplasia of skene ducts in a diethylstilbestrol-exposed woman. Am J Clin Pathol 2015; 144: 790–95. doi: 10.1309/AJCPVZ0QNLUO7OFE 26486744

[4] Clement PB, Benedet JL . Adenocarcinoma in situ of the vagina: a case report. Cancer 1979; 43: 2479–85. doi: 10.1002/1097-0142(197906)43:6<2479::aid-cncr2820430645>3.0.co;2-o 455232

[5] Dhorepatil B, Ladda DK, Rapol AU . Rare case of primary mucinous adenocarcinoma of vagina. J Cancer Res Ther 2013; 9: 514–16. doi: 10.4103/0973-1482.119366 24125996

[6] Turashvili G, Park KJ . Cervical glandular neoplasia: classification and staging. Surg Pathol Clin 2019; 12: 281–313. doi: 10.1016/j.path.2019.01.002 31097105

[7] Hiniker SM, Roux A, Murphy JD, Harris JP, Tran PT, Kapp DS, et al . Primary squamous cell carcinoma of the vagina: prognostic factors, treatment patterns, and outcomes. Gynecol Oncol 2013; 131: 380–85. doi: 10.1016/j.ygyno.2013.08.012 23954572

[8] Lima M, Rio G, Horta M, Cunha TM . Primary vaginal malignancies: a single oncology centre experience. J Obstet Gynaecol 2019; 39: 827–32. doi: 10.1080/01443615.2019.1579786 31020870

[9] Siegelman ES, Outwater EK, Banner MP, Ramchandani P, Anderson TL, Schnall MD . High-Resolution MR imaging of the vagina. Radiographics 1997; 17: 1183–1203. doi: 10.1148/radiographics.17.5.9308110 9308110

[10] Liu Y, Xi-Ling Z, Ji-Qi Y, et al . Non-surgical treatment of calcified gastric signet ring cell carcinoma: a case report. J Oncol 2017; 458–60.

[11] Ling-Ying C, Chun-Yan L . Clinicopathological features and CT diagnosis of gastric signet ring cell carcinoma. Chinese Journal of Basic and Clinical Surgery 2022; 376–81.

[12] Zhen-Hui L, Xing-Xiang D, De-Pei G, et al . Comparison of CT manifestations between colorectal primary signet ring cell carcinoma and adenocarcinoma. China Journal of Medical Imaging 2015; 834–38.

[13] Jiang H-P, Wei Q-Z, Guo S-Q . A case of primary signet ring cell carcinoma of the cervix. Guangdong Medical Journal 2014; 35: 1212.

